# Individual host variation in single-cell responses to enteroaggregative *Escherichia coli* infection modeled with human colon organoids

**DOI:** 10.1128/msphere.00758-25

**Published:** 2026-08-11

**Authors:** Ling Qiu, Tajhal D. Patel, Hannah E. Jones, Cristian Coarfa, Anthony W. Maresso

**Affiliations:** 1 Department of Molecular Virology and Microbiology, Baylor College of Medicine 3989, Houston, Texas, USA; 2 Pediatric Oncology, Baylor College of Medicine 3989, Houston, Texas, USA; 3 Department of Molecular and Cellular Biology, Baylor College of Medicine 3989, Houston, Texas, USA; 4 Dan L Duncan Comprehensive Cancer Center, Baylor College of Medicine 3989, Houston, Texas, USA; 5 Center for Precision Environmental Health, Baylor College of Medicine 3989, Houston, Texas, USA; 6 TAILФR Labs, Baylor College of Medicine 3989, Houston, Texas, USA; University of Michigan Medical School, Ann Arbor, Michigan, USA

**Keywords:** *Escherichia coli*, host-pathogen interactions, infection response, human intestinal organoids, epithelial cells, innate immunity, host susceptibility, bioinformatics, host heterogeneity, single-cell RNA sequencing, enteric infection, bacterial infection

## Abstract

Enteroaggregative *Escherichia coli* (EAEC) is a leading cause of diarrhea worldwide. Interestingly, EAEC disease outcomes vary dramatically among infected individuals, ranging from asymptomatic colonization to acute and chronic diarrhea. While previous research has focused on EAEC virulence factors, emerging evidence suggests that host genetic and cellular background may be equally important in determining disease susceptibility. To understand host-driven disease heterogeneity, we performed single-cell RNA sequencing on human colon organoids (HCOs) derived from three healthy adult donors following EAEC 042 infection. Surprisingly, despite comparable EAEC adherence across donors, we observed striking donor-specific responses to infection at multiple levels. A total of seven distinct colonic epithelial cell clusters were identified, with striking donor-specific baseline compositions. Following infection, the HCO donors exhibited three distinct response phenotypes evident in both cell type remodeling and gene regulation: hyperresponsive, intermediate, and non-responsive. We further observed extensive donor-specific cell-cell communication networks at baseline and after EAEC infection. This study suggests that the heterogeneity in host epithelial response to EAEC infection extends to the single-cell level. While the colonic regions from which the HCOs were derived represent a potential confounding factor, the magnitude of organoid-to-organoid variation exceeded previously reported regional differences, suggesting intrinsic host factors as a crucial part of response heterogeneity. These findings establish a paradigm for understanding infectious disease susceptibility through the human organoid model system.

**IMPORTANCE:**

Understanding why similar pathogen exposures produce vastly different clinical outcomes is critical for combating infectious diseases. By conducting single-cell RNA sequencing on human colon organoids derived from three different donors, we show that each donor possesses unique intrinsic cellular compositions and distinct transcriptomic responses to enteroaggregative *Escherichia coli* (EAEC) infection. These findings challenge the traditional pathogen-focused paradigm and highlight the critical role of host factors in infectious disease outcomes. This study also validates human organoids as a powerful platform for studying host heterogeneity, which may help predict disease susceptibility and optimize treatment for diverse patient populations in the future.

## INTRODUCTION

Historically, research on bacterial pathogenesis has largely focused on the pathogens. In particular, studies in the field have emphasized bacterial virulence factors involved in nutrient acquisition, colonization, toxin production, and immunomodulation (1). While such research has led to successful vaccines and anti-virulence therapeutic strategies, it often overlooks an equally critical component of disease: the host. Host factors—including age, sex, genetics, microbiome, immune status, and comorbidities—play a central role in determining susceptibility to bacterial infection and disease severity (2). Leveraging host biology offers great potential for tackling infections and addressing the growing threat of antibiotic resistance. However, our understanding of host contributions to pathogenesis remains severely limited.

Enteroaggregative *Escherichia coli* (EAEC) is a group of enteric bacteria that cause acute and persistent diarrhea in children (3, 4), travelers (5), and immunocompromised patients (6). Pediatric EAEC infections are particularly problematic due to its association with malnourishment and impaired growth (7), even in the absence of overt diarrhea symptoms (8, 9). Despite its global impact and serious sequelae, treatment options for EAEC remain limited to ineffective antibiotics, largely due to widespread multidrug resistance observed across diverse geographical regions (10) and in over 70% of clinical isolates (11). To combat the emergence of highly virulent hybrid strains (12), a deeper understanding of the molecular mechanisms underlying EAEC pathogenesis is essential. Major EAEC virulence factors include aggregative adherence fimbriae, which mediate adhesion to and colonization of the intestinal mucosa (13); the protein involved in colonization (Pic), which degrades mucin (14); and the plasmid-encoded toxin (Pet), which induces cytotoxicity (15). Nevertheless, clinical manifestations of EAEC infection range widely from asymptomatic carriage to acute and chronic diarrhea, a variability not fully explained by EAEC virulence factors or bacterial load (16, 17). Furthermore, human volunteer studies have shown that the ability of EAEC to colonize and cause diarrhea varies depending on the host (18). These findings further highlight the importance of the host in determining infection outcomes and underscore the urgent need to better understand host biology in the context of EAEC pathogenesis.

Using EAEC as a pathogen to model host differences, we aim to investigate host-specific variation in infection response using human colon organoids (HCOs). HCOs are non-transformed, tissue stem cell-derived models that, upon *in vitro* differentiation, closely recapitulate the cellular composition and functional characteristics of the mature human colon epithelium (19). Crucially, HCOs retain the genetic diversity of their donors, offering a unique opportunity to examine how host genetic background influences infection outcomes. Previous research from our group (20, 21) and others (22) has established HCOs as a robust model for studying EAEC infection, revealing both segment- and host-specific patterns of colonization. However, while bacterial binding and colonization patterns have been explored, differences in the host response to EAEC infection across donors remain largely unexamined. Recent studies have leveraged single-cell RNA sequencing (scRNA-seq) to dissect cell type-specific responses to pathogens within the intestinal epithelium (23–25). However, most scRNA-seq studies involving human organoids treat donor variation as a source of biological noise, averaging out potentially meaningful differences. By harnessing the inherent genetic diversity of HCO donors, we investigated donor-specific infection responses at single-cell resolution. This approach helps uncover epithelial cell subtypes and pathways that confer protection against EAEC, advancing our understanding of host determinants in bacterial pathogenesis. Here, we describe substantial host-specific responses at the single-cell level despite uniform infection conditions and pathogen strain.

## RESULTS

**Fig 1:**
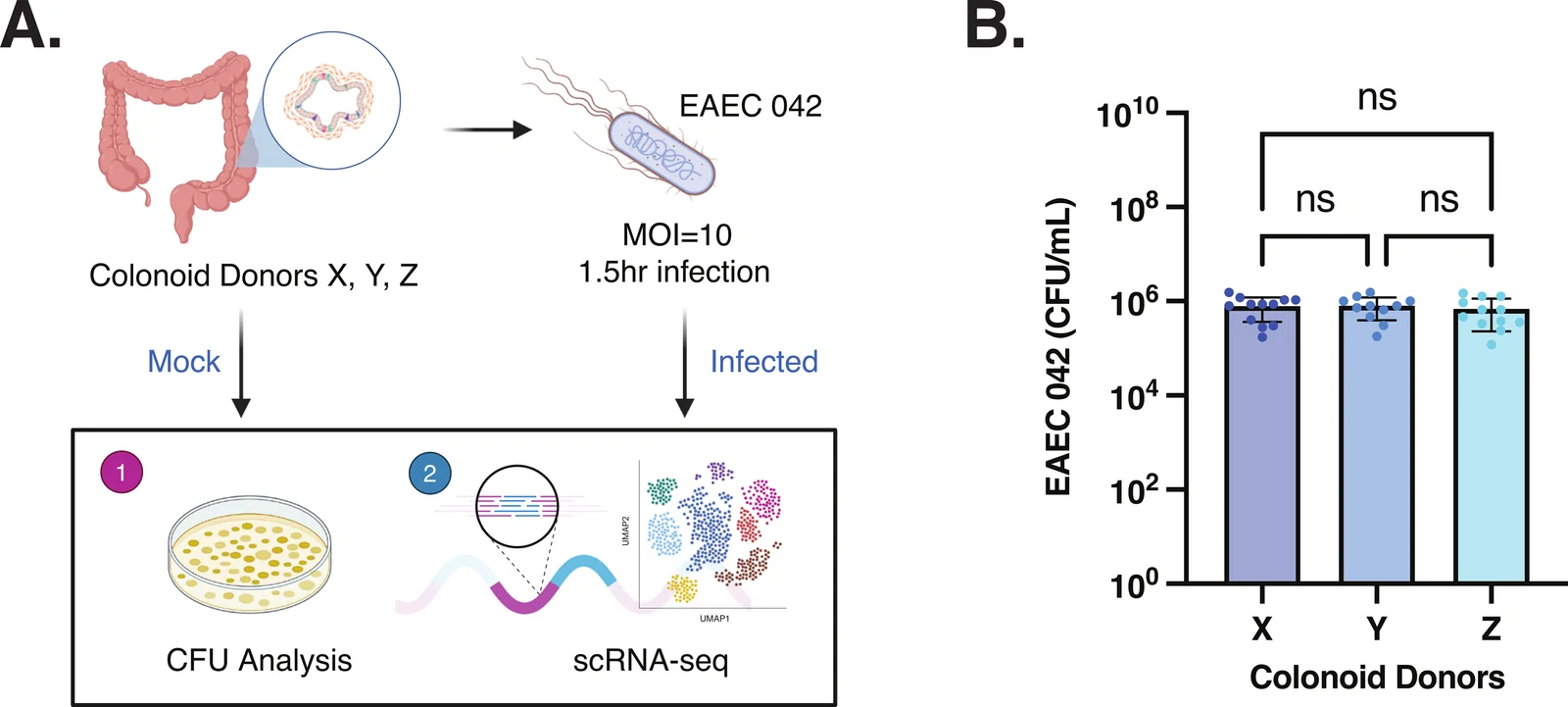
Colonoid scRNA-seq experiment design. (**A**) Schematic of scRNA-seq and bacterial quantification on EAEC-infected colonoids. Briefly, colon organoids derived from intestinal crypts of three healthy donors were plated as monolayers on a 96-well plate and differentiated for 4 days. Colonoid monolayers were either mock infected with fresh differentiation media or with log-phase EAEC 042 at MOI = 10 for 1.5 h at 37°C in a humidified incubator. After infection, colonoid monolayers were washed to remove unbound bacteria, collected, and separated into single-cell suspensions, and then sequenced via the 10x platform. Additional colonoids were collected and plated on LB agar to quantify bacterial loads. (**B**) Bar plot depicting EAEC 042 load (CFU/mL) adhered to colonoid monolayers after 1.5 h of infection (ns, not significant, *P* > 0.05).

Building off previous results (20, 21) that the response to EAEC infection is driven largely by differences in the host, we sought to determine if such observations extend to individual cells within patient-derived organoid lines. To test this idea, we selected three HCO lines from our biobank and performed EAEC infection, followed by single-cell RNA sequencing. Organoids from the colon were used because EAEC shows a strong large bowel disease presentation (26, 27). For clarity, we refer to these lines as X, Y, and Z and report the donor characteristics in Table S1. The experimental workflow is illustrated in Fig. 1A. In brief, colonoid monolayers were differentiated for 4 days, then either mock-infected or infected with EAEC strain 042 for 1.5 h. Following infection, the monolayers were then dissociated into single-cell suspensions and subjected to single-cell RNA sequencing using the 10x Genomics platform. To ensure comparable levels of infection across lines, we quantified bacterial burden by determining colony-forming units (CFUs) adhering to the monolayers after the washing step. As shown in Fig. 1B, there were no significant differences in bacterial adhesion among the three donor lines, indicating that any variation in host response is likely due to intrinsic differences in host genotype or phenotype, rather than differences in bacterial load.

### Cell type annotation and donor intrinsic differences

**Fig 2:**
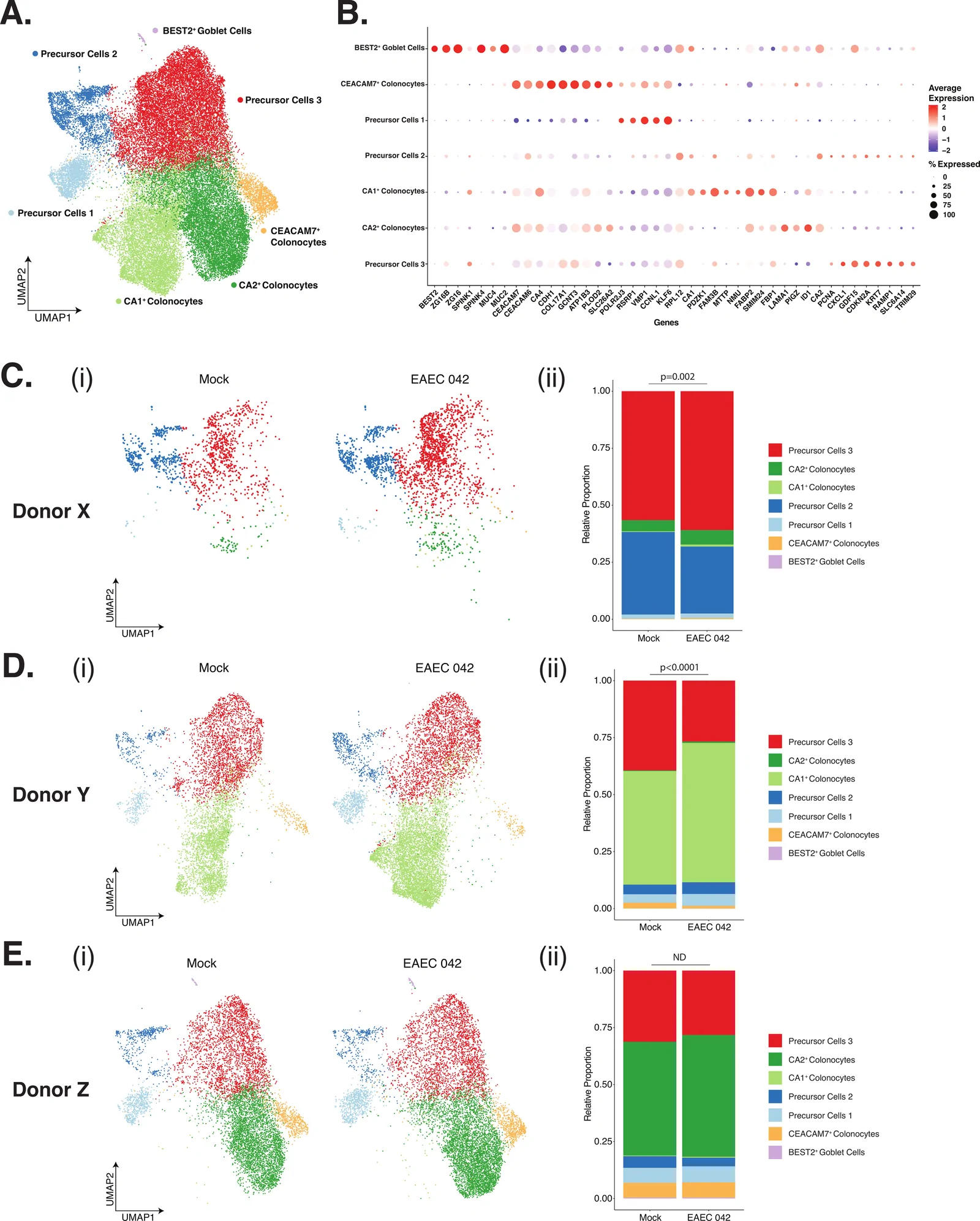
Donor-specific cell type distribution and changes induced by EAEC infection. (**A**) UMAP showing seven cell clusters at Leiden resolution 0.4. (**B**) Dot plot showing relative gene expression of cell cluster markers. (**C**) Cell cluster distribution of donor X under mock- or EAEC-infection conditions, visualized using UMAP (i), with the relative proportion of each cluster presented in the accompanying bar plot (ii). (**D**) Same as panel **C** but for donor Y. (**E**) Same as panel **C** but for donor Z. Change in cell composition between Mock and EAEC is assessed using a chi-square test.

A critical first step before evaluating EAEC-induced transcriptional changes is to accurately annotate the cellular composition of the population. After QC filtering, a total of 39,351 cells remained for downstream data analysis. Filtered scRNA-seq data, including all mock and infected cells, were first processed using Scanpy and Seurat. Clustering with the Leiden algorithm (28) revealed seven distinct cell populations, visualized by uniform manifold approximation and projection (UMAP) (Fig. 2A). We next annotated these clusters as seven different colonic epithelial cell subtypes, including colonocytes, goblet cells, and precursor cells (Fig. 2B). To define the developmental relationships among the precursor populations, we applied Slingshot pseudotime analysis (Fig. S1A and B). This analysis suggested a differentiation trajectory originating from precursor cells 1, the least differentiated population, progressing through precursor cells 2 and precursor cells 3, before diverging into four differentiated populations: *BEST2^+^* goblet cells, *CA2^+^* colonocytes, *CA1^+^* colonocytes, and *CEACAM7^+^* colonocytes. The naming of precursor cells 1, 2, and 3 reflects their inferred order along this developmental trajectory. Each precursor cluster expressed distinct combinations of proliferative and epithelial lineage markers (Fig. 2B). Precursor cells 1 were enriched for the progenitor marker *POLR2J3* and early enterocyte markers *RSRP1*, *VMP1*, and *KLF6*. Precursor cells 2 expressed intestinal stem cell markers *RPL12* and *PCNA*, alongside the colonocyte marker *CA2*. Interestingly, precursor cells 3 co-expressed proliferative markers (*RPL12*, *GDF15*, *PCNA*), goblet cell markers (*MUC4*, *ZG16B*, *SPINK1*, *RAMP1*), tuft cell markers (*KRT7*, *MYO1B, TRIM29*, *SLC6A14*). This population also showed high expression of chemokine-encoding genes, including *CXCL1*, *CXCL8*, *CXCL3*, and *CXCL16*. All three colonocyte populations expressed canonical enterocyte markers *FABP2* and *SLC26A2* but were distinguished by cluster-specific signatures. *CA1^+^* colonocytes expressed *PDZK1*, *FAM3B*, *MTTP*, and *NMU*; *CA2^+^* colonocytes expressed *LAMA1*, *LINC00668*, *GABRA2*, and *RPS4Y1*; *CEACAM7^+^* colonocytes expressed *KCNQ1OT1*, *BTNL8*, *SLC30A10*, and *PLOD2*.

Next, we stratified the seven colonic epithelial cell clusters by donor and observed distinct cell type distributions across the three colonoid lines (Fig. S1C and D). Donor X exhibited a predominance of undifferentiated populations, with over 90% of cells classified as either precursor cells 2 or precursor cells 3. In contrast, differentiated cell types were enriched in donors Y and Z, with *CA1^+^* colonocytes being the major population in donor Y, and *CA2^+^* colonocytes dominating in donor Z. Notably, *CA1^+^* colonocytes were largely unique to donor Y, while *CA2^+^* colonocytes and *BEST2^+^* goblet cells were almost exclusively observed in donor Z, highlighting striking donor-specific intrinsic differences in epithelial cell composition. These observations suggest that tissue stem cell-derived colon organoids may retain donor-specific characteristics that reflect the genetic landscape of the original tissue.

### Infection-driven differences in cell clusters

Evaluating changes in cell type distribution induced by EAEC 042 infection for each donor, we were surprised to find significant host-specific changes (Fig. 2C through E and Fig. S1E and F). Among the three donors, donor Z appeared relatively resistant to EAEC colonization (Fig. 2E), exhibiting no significant changes in cell type composition following infection—classifying this donor as a non-responder to EAEC infection. In contrast, both donors X and Y displayed significant alterations in cluster distribution after bacterial exposure (Fig. 2C and D), therefore classifying as responders to EAEC infection. In donor X (Fig. 2C), infection was associated with an increase in precursor cells 3, a population enriched in immune-activating transcripts. Donor Y (Fig. 2D), on the other hand, showed an enrichment of CA1^+^ colonocytes and a depletion of precursor cells 3, potentially reflecting enhanced epithelial function, such as pH regulation by mature colonocytes. While the mechanistic basis for these shifts remains to be elucidated, our findings indicate that organoid donor identity not only influences susceptibility to EAEC adherence (20, 21), a hallmark of EAEC pathogenesis, but also drives distinct transcriptional and cellular remodeling during infection at the single-cell level.

### Infection-driven differences in gene regulation

**Fig 3:**
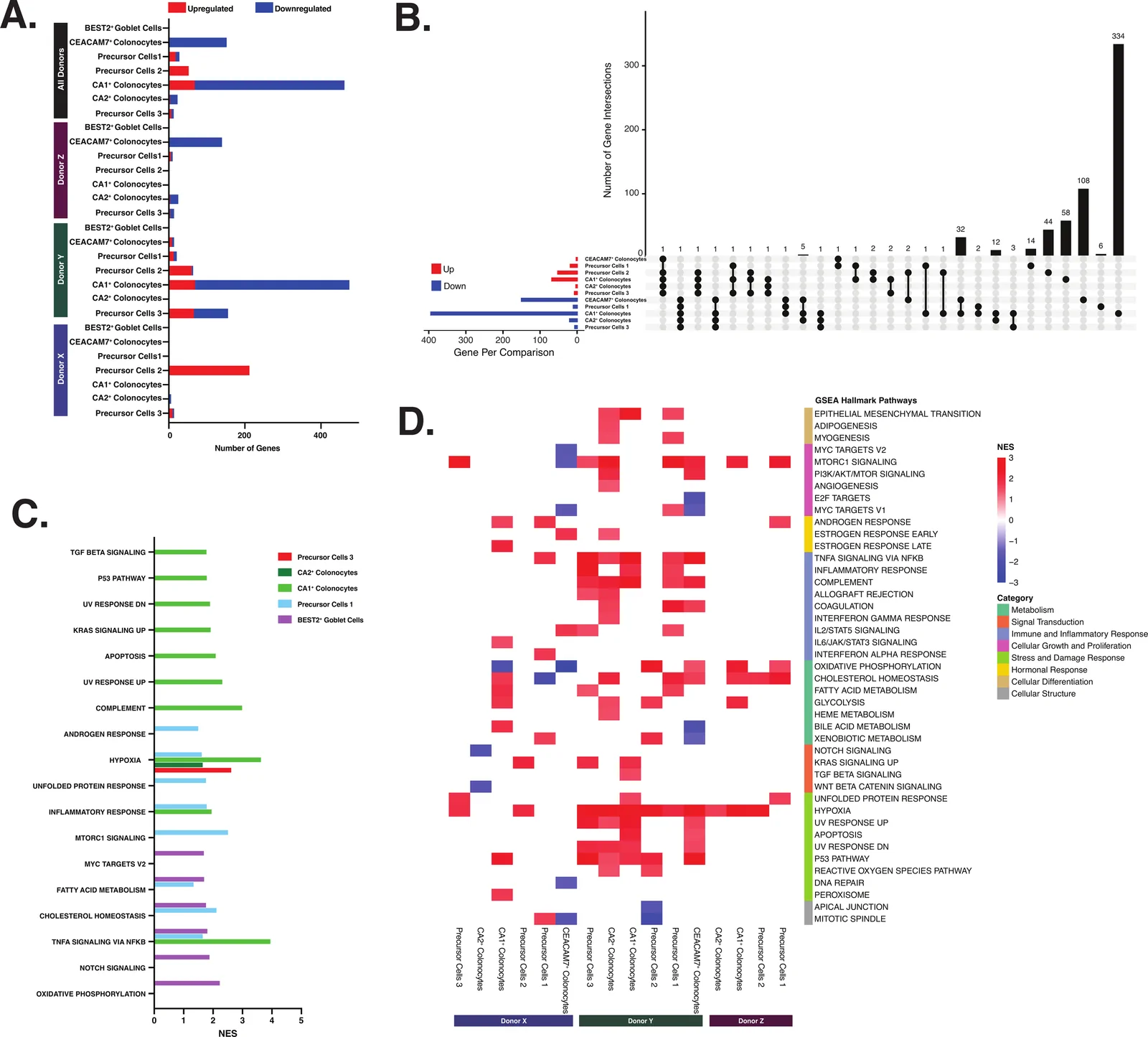
Donor-specific gene and pathway regulation induced by EAEC infection. (**A**) Stacked bar diagram showing number of DEGs between EAEC-infected and mock-infected colonoids (FDR < 0.1, fold change exceeding 1.25×). (**B**) UpSet plot showing overlapping DEGs across different cell clusters using pooled donors. (**C**) Bar plot showing infection-enriched hallmark pathways for different cell clusters, using gene set enrichment analysis (GSEA). Normalized enrichment scores (NES) are presented. (**D**) Heatmap showing infection-enriched hallmark pathways for each cell cluster within each donor. NES are shown for enriched pathways.

Since each donor displayed a “personalized” cell distribution upon infection, we next asked whether the cell populations within each donor also differentially regulate gene expression in response to EAEC infection. To address this, we performed a differentially expressed genes (DEG) analysis (Mann-Whitney-Wilcoxon, FDR < 0.1, fold change exceeding 1.25×). As an initial step, we confirmed that the observed differences could not be attributed to baseline gene expression variation among donors (Fig. S2). Overall, a greater number of genes were downregulated than upregulated (Fig. 3A). Interestingly, the infection-induced DEGs of each donor not only differed in regulatory directions but also in the driving cell types: donor X showed predominantly upregulated DEGs driven by precursor cells 2; donor Y exhibited mixed regulation dominated by *CA1*^+^ colonocytes; donor Z demonstrated predominantly downregulated DEGs driven by *CEACAM7*^+^ colonocytes. We next examined overlap among DEG sets across clusters computed by pooling all the donors (Fig. 3B). We found that most overlapping DEGs were shared among the three mature colonocyte populations. For example, *CA1^+^* and *CEACAM7*^+^ colonocytes shared 32 downregulated DEGs. *CA2^+^* and *CA1^+^* colonocytes also shared 12 downregulated DEGs. These findings highlight that although donors harbor different proportions of colonocyte subtypes, these cells share conserved transcriptional programs to combat EAEC infection. Surprisingly, despite these overlapping transcriptional responses, most DEGs are unique to each cluster. For example, *CA1^+^* colonocytes alone harbor >300 unique DEGs. This suggests that epithelial subpopulations have evolved specialized roles in pathogen defense, with the distinct cellular architecture of each donor contributing to personalized antimicrobial response strategies.

Next, to investigate the molecular pathways altered by EAEC infection, we performed GSEA hallmark pathway analysis for the infection response. Overall, we observed a general upregulation of hallmark pathways (Fig. 3C), primarily driven by *CA1^+^* colonocytes, precursor cells 1, and *BEST2^+^* goblet cells. Notably, most upregulated pathways were associated with immune and inflammatory responses, as well as stress and damage responses. For instance, hypoxia pathways were elevated in four clusters, and TNFɑ signaling via NF-κB pathways was enhanced in three clusters. This likely reflects universal metabolic stress induced by EAEC virulence factors.

When further examining GSEA hallmark pathway regulation by donor (Fig. 3D), we found striking variability in their responses to EAEC infection. Donor Y exhibited the strongest response, with widespread upregulation in pathways related to cellular differentiation, immune and inflammatory signaling, metabolism, and stress responses. Such a “hyperresponsive” phenotype in both cell remodeling and pathway regulation suggests an active epithelial defensive response against pathogen challenge in donor Y. In contrast, donor Z showed the weakest response, with no detectable changes in cellular differentiation, immune response, signal transduction, or structural pathways. This is consistent with the “non-responsive” phenotype observed in the cell composition analysis (Fig. 2). It is uncertain if this relates to the selective upregulation of *CA2*^+^ colonocytes as a result of the higher catalytic activity of CA2 in pH regulation. Mucin secretion by *BEST2*^+^ goblet cells in donor Z may also help maintain homeostasis and protect the host from epithelial damage. Donor X displayed an intermediate level of regulation in GSEA hallmark pathways. This “intermediate responsive” phenotype, characterized by expansion of immune-activated precursor cells 3, may represent a balanced response that mounts antibacterial defense while minimizing tissue inflammatory damage.

Beyond overall pathway differences, the same cell populations exhibited distinct response patterns across donors. For example, *CA2^+^* colonocytes exhibited consistent upregulation of hallmark pathways in donors Y and Z but displayed downregulation of signal transduction pathways in donor X, specifically Notch and Wnt/β-catenin signaling. Similarly, *CEACAM7^+^* colonocytes in donor X showed broad pathway downregulation, including in MYC signaling, mTORC1 signaling, oxidative phosphorylation, and DNA repair. However, this cell cluster showed mainly pathway upregulation in donor Y and no detectable changes in donor Z. Together, these findings underscore that EAEC elicits highly donor-specific gene regulation events, with variability evident not only across individuals but also at the level of individual cell populations.

### Donor-specific cell-cell communication in response to infection

**Fig 4:**
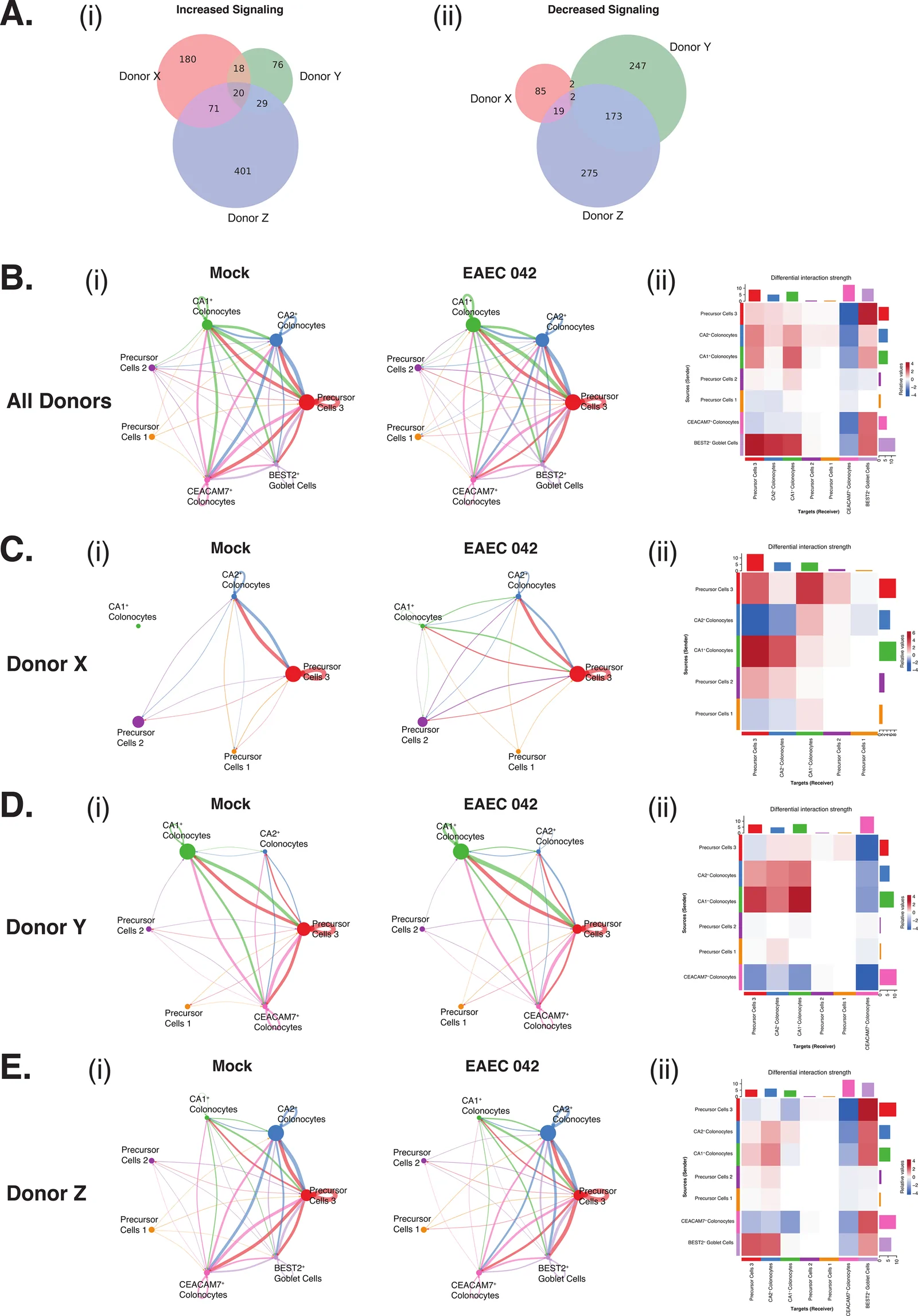
CellChat ligand-receptor analysis. (**A**) Venn diagrams showing the numbers of (i) increased and (ii) decreased signaling interactions caused by EAEC infection in each donor. (**B**) (i) Circle plots showing interactions between clusters with or without EAEC infection for all cells. (ii) Heatmap summarizes changes in cell-cell interactions for each pair of source and target clusters. (**C**) Same as panel **B** but for donor X. (**D**) Same as panel **B** but for donor Y. (**E**) Same as panel **B** but for donor Z.

CellChat is a computational framework that enables the characterization of intercellular communication dynamics by analyzing ligand-receptor pairings in complex single-cell datasets (29). Using this approach, we identified >1,500 significantly altered signaling interactions following EAEC infection (Fig. 4A). Among the three donors, donor Z exhibited the largest number of both upregulated and downregulated interactions. In contrast, donor Y displayed the fewest increased signaling events, while donor X exhibited the fewest decreased events. Strikingly, given the high numbers of total upregulated and downregulated signaling events, only a limited number of interactions were commonly altered across all donors. Specifically, there were 20 shared upregulated and merely 2 shared downregulated signaling interactions. These results suggest that individual donors exhibit not only distinct cell-specific responses to infection, but also different functional consequences of these responses.

When pooling all donors, circular interaction plots revealed that in both mock- and EAEC-infected states, the majority of intercellular communications were mediated by precursor cells 3, *CA2*^+^ colonocytes, and *CA1*^+^ colonocytes (Fig. 4B-i). Among these, precursor cells 3 were the most active signaling population, engaging in bidirectional communication with goblet cells and all mature colonocyte subtypes. In the uninfected state, precursor cells 1 were unique in exclusively initiating signaling without receiving input from other clusters, and both precursor cells 1 and 2 lacked intra-cluster communication. Upon infection, the most prominent changes were observed in crosstalk between precursor cells 3 and *BEST2^+^* goblet cells (Fig. 4B-ii). Goblet cells also displayed enhanced intra-cluster signaling and increased communication with both *CA1^+^* and *CA2^+^* colonocytes. Conversely, *CEACAM7*^+^ colonocytes were the major targets of downregulated communication, particularly reducing interactions initiated by precursor cells 3. Notably, despite their overall reduction in signaling as targets, *CEACAM7*^+^ colonocytes increased communication with *BEST2*^+^ goblet cells after infection. Precursor cells 1 also began receiving signaling from precursor cells 3 and *CA2*^+^ colonocytes upon infection, although overall, precursor cells 1 and 2 remained relatively stable in their communication landscape. Collectively, these findings underscore the central role of precursor cells 3 in coordinating host responses to EAEC infection, potentially due to its high abundance and expression of immune-activating genes.

Having established the overall landscape of cell-cell communication across all samples, we next examined how these signaling dynamics varied among individual donors (Fig. 4C through E). Donor X exhibited the simplest network of signaling events (Fig. 4C). In this donor, the strongest interactions occurred between precursor cells 3 and *CA2*^+^ colonocytes, regardless of infection status. Under mock conditions, *CA1*^+^ colonocytes did not participate in any signaling. However, following infection, *CA1*^+^ colonocytes received signaling input from all clusters—most prominently from precursor cells 3—and initiated communication with all clusters except precursor cells 1, including strong intra-cluster signaling. precursor cells 3 and *CA2*^+^ colonocytes showed reduced signaling to precursor cells 1, leaving the latter with only outward communication. Interestingly, although *CEACAM7^+^* colonocytes were present in donor X (Fig. 2C), they were not engaged in any cell-cell signaling activities. In donor Y (Fig. 4D), the most robust interactions were observed between precursor cells 3 and *CA1*^+^ colonocytes, with the stress from EAEC infection further amplifying this communication. Consistent with global analyses, donor Y exhibited more downregulated than upregulated signaling events after infection (Fig. 4A), the majority of which were mediated by *CEACAM7^+^* colonocytes. Donor Z displayed the most complex communication network and the highest number of altered signaling interactions (Fig. 4A and E) despite being a non-responder in transcriptional responses. In this donor, the strongest baseline interactions occurred among precursor cells 3, *CA2*^+^ colonocytes, *CEACAM7^+^* colonocytes, and *BEST2*^+^ goblet cells. EAEC infection resulted in marked increases in bidirectional signaling between precursor cells 3 and *BEST2*^+^ goblet cells, alongside pronounced bidirectional decreases between precursor cells 3 and *CEACAM7^+^* colonocytes. Similar to donor Y, *CEACAM7^+^* colonocytes mediated the majority of downregulated interactions, yet their communication into *BEST2*^+^ goblet cells increased post-infection.

Taken together, these results demonstrate that while precursor cells 3 consistently serve as a central signaling hub across donors, each donor possesses a unique baseline communication landscape that is differentially remodeled by EAEC infection. These donor-specific communication profiles highlight the remarkable heterogeneity of host responses at the intercellular level within patient-derived colonoids.

### Donor-specific ligand-receptor response in CEACAM and GDF pathways

With the total cell-cell interactions showing striking donor variability both at baseline and in infection-induced changes, we next sought to investigate specific CellChat pathway interactions involved in EAEC infection. Carcinoembryonic antigen-related cell adhesion molecules (CEACAMs) are immunoglobulin-related glycoproteins that regulate key cellular processes, including cell adhesion, differentiation, proliferation, and survival (30). Altered CEACAM expression has been implicated in gastrointestinal diseases, such as inflammatory bowel disease and colon cancer. Given the importance of CEACAM molecules in maintaining intestinal epithelial homeostasis, we investigated CEACAM-mediated communication across the seven colon epithelial cell subtypes. In this pathway, CEACAM1 functions as the ligand and CEACAM5 as the receptor. While both are broadly expressed in colon tissue, CEACAM1 is enriched in absorptive colonocytes, whereas CEACAM5 is more highly expressed in mucus-secreting colonocytes (31).

**Fig 5:**
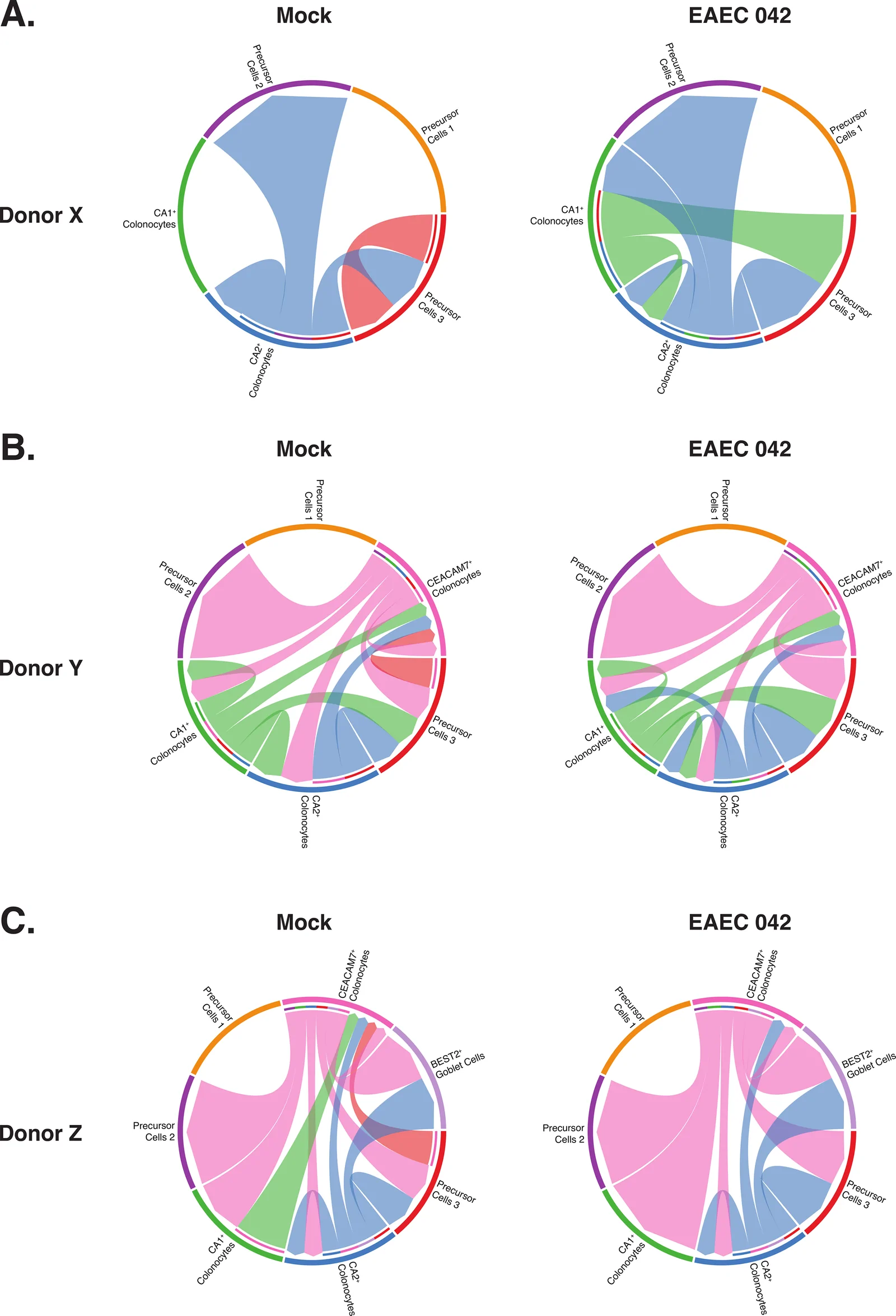
CellChat CEACAM pathway analysis chord diagrams showing the CEACAM pathway ligand-receptor interaction among colonoid cell clusters in donor X (**A**), donor Y (**B**), and donor Z (**C**) under mock- or EAEC-infected conditions.

In donor X (Fig. 5A), under mock-infected conditions, CEACAM signaling was primarily initiated by *CA2^+^* colonocytes and received by the same cell type, as well as precursor cells 2 and 3. Following EAEC 042 infection, communication from *CA2^+^* colonocytes increased toward all these populations, and additional bidirectional signaling emerged between *CA2^+^* and *CA1^+^* colonocytes. Meanwhile, self-signaling within precursor cells 3 disappeared and was replaced by input from *CA1^+^* colonocytes. In donor Y (Fig. 5B), *CEACAM7^+^* and *CA1^+^* colonocytes dominated signaling under both mock and infected conditions. *CEACAM7^+^* colonocytes communicated with all cell types except precursor cells 1, while *CA1^+^* colonocytes signaled broadly except to precursor cells 1 and 2. Unlike donor X, where signaling largely increased post-infection, donor Y exhibited a mixed pattern of gains and losses. Specifically, *CA2^+^* colonocytes strengthened communication with *CEACAM7^+^* colonocytes and precursor cells 3, while also establishing new interactions with *CA1^+^* colonocytes and their own population. By contrast, signaling from precursor cells 3 to *CEACAM7^+^* colonocytes was lost. In donor Z (Fig. 5C), *CEACAM7^+^* colonocytes were the primary signaling cluster, targeting nearly all clusters except precursor cells 1. *CA2^+^* colonocytes were also highly active, signaling to their own cells, precursor cells 3, *CEACAM7^+^* colonocytes, and *BEST2*^+^ goblet cells. EAEC infection further amplified these communications in donor Z, while interactions from precursor cells 3 and *CA1^+^* colonocytes to *CEACAM7^+^* colonocytes were lost. Taken together, *CA2^+^* colonocytes consistently acted as the major CEACAM signaling initiators across all three donors, while *CEACAM7^+^* colonocytes were both initiators and receivers in donors Y and Z. Notably, precursor cells 3 universally lost their signaling capacity after infection. Despite these commonalities, each donor exhibited distinct communication patterns within the CEACAM pathway. This suggests potential variations in CEACAM expression in different colon epithelial clusters that can influence barrier function and lead to donor-specific epithelial cell adhesion communications in response to infection-induced stress.

We next examined the growth and differentiation factor (GDF) pathway, in which GDF15 acts as the ligand and transforming growth factor beta receptor type 2 (TGFBR2) as the receptor. GDF15 is an inflammation-induced hormone, elevated in bacterial infections, such as sepsis, where it has protective effects through enhancing tissue tolerance to inflammatory damage (32). Ligand activation of TGFBR2 triggers canonical SMAD-mediated TGF-β signaling (33), which reduces bacterial burden and mitigates tissue damage (34). Thus, donor-specific GDF ligand-receptor interactions may provide insight into variability in bacteria-induced host inflammatory responses.

**Fig 6:**
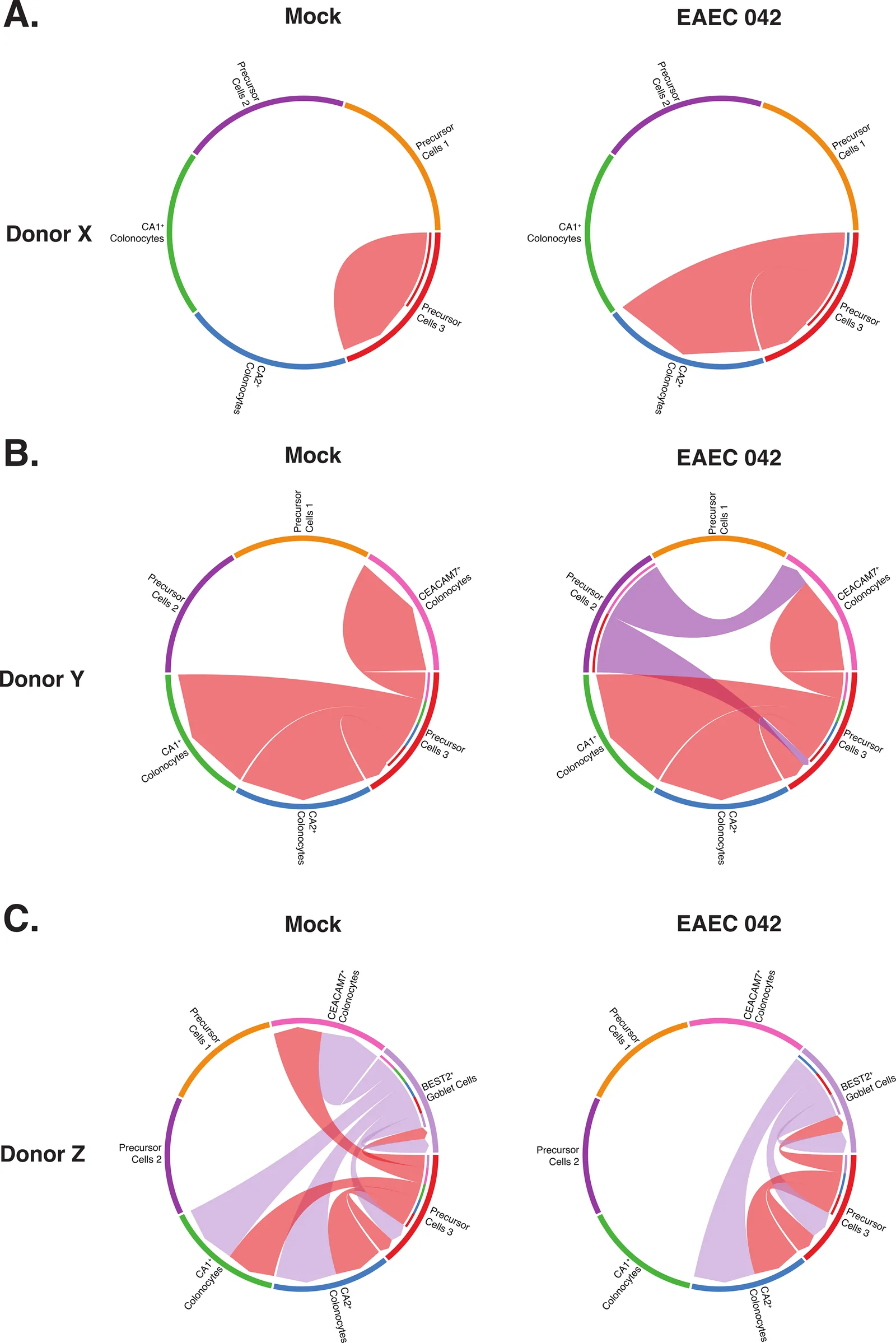
CellChat GDF pathway analysis chord diagrams showing the CEACAM pathway ligand-receptor interaction among colonoid cell clusters in donor X (**A**), donor Y (**B**), and donor Z (**C**) under mock- or EAEC-infected conditions.

In donor X (Fig. 6A), GDF signaling occurred only within precursor cells 3 under mock conditions, but intensified after infection, with new interactions directed toward *CA2^+^* colonocytes. In donor Y (Fig. 6B), precursor cells 3 also dominated signaling under both mock and infected conditions, targeting their own cluster, as well as all mature colonocyte populations. Infection further increased signaling, introducing new interactions such as communication from precursor cells 2 to *CEACAM7^+^* colonocytes and precursor cells 3. In donor Z (Fig. 6C), signaling was primarily initiated by precursor cells 3 and *BEST2*^+^ goblet cells, targeting most populations except precursor cells 1 and 2. Unlike the other donors, donor Z showed a pronounced loss of signaling after infection. Notably, *CA1^+^* and *CEACAM7^+^* colonocytes no longer act as receptors of interaction. Overall, precursor cells 3 consistently served as the major GDF ligand source across donors, highlighting its important role in mediating host inflammatory response. Since both the recipient populations and the infection-induced changes in the GDF pathway varied considerably among donors, these findings suggest that the magnitude of inflammatory response to enteric infections may be dependent on donor cell type distributions and receptor expression profiles. In conclusion, our analysis of cell-cell communication profiles in CEACAM and GDF pathways further underscores donor-specific differences in epithelial responses to bacterial infection.

## DISCUSSION

The present study leverages the advanced technology of single-cell transcriptomics and the emerging tool of human organoid model systems to demonstrate dramatic host variation in intestinal epithelial responses to EAEC infection. Building upon previous observations that EAEC infection displays a segment- and donor-dependent trend (20, 21), our study shows that such heterogeneity extends beyond bacterial adherence to cell type composition (Fig. 2), transcriptional response (Fig. 3), and intercellular communication (Fig. 4 to 6). We identified three distinct EAEC infection response phenotypes: hyperresponsive (donor Y), intermediate responsive (donor X), and non-responsive (donor Z). Our study emphasizes the urgent need to consider host genetic diversity when studying infectious disease mechanisms and developing therapeutic interventions.

Variation in data generated using human organoids has been an active point of discussion within the field. It is a double-edged sword: while such variation raises concerns regarding rigor and reproducibility (35), it also reflects the inherent heterogeneity of the model. Previous studies of human intestinal organoids have demonstrated that they retain the physiological characteristics of their source tissue (36, 37) and preserve donor-specific phenotypes, including age-related differences (38). One study characterizing donor variations in human intestinal organoids reported comparable differentiation patterns across three donors but identified differences in metabolic activity and hormone secretion (39). To our knowledge, our work is the first to show such dramatic donor-specific differences in single-cell composition and bacterial infection response among healthy adult colon organoids. The striking heterogeneity in baseline cellular composition observed across donors likely underlies differences in infection susceptibility and cellular communication changes. This donor-dependent response may explain why some individuals develop severe EAEC-associated diarrhea while others remain asymptomatic despite similar bacterial exposure and colonization.

This study has some important clinical implications. First, the profound donor-specific differences we observed are important for considering precision medicine approaches when treating bacterial infections. The identification of distinct host response phenotypes highlights the importance of tailoring therapeutic interventions to individual needs. For example, hyperresponsive patients might require anti-inflammatory treatments, while non-responsive patients might benefit from a boost in antimicrobial response. Additionally, the vastly different host response despite comparable bacterial adherence suggests that current diagnostic approaches based on bacterial burden may not accurately predict disease severity. Instead, transcriptional signatures might serve as better prognostic indicators. Lastly, our work demonstrates the great potential of using human organoids to discover donor-specific mechanisms of pathogen defense. It is therefore important to harness and expand the biodiversity in organoid biobanks for more systemic studies that aim to identify intrinsic host factors that confer susceptibility or resistance to infection.

Our study has several limitations that should be considered when interpreting the findings. First, an important caveat is that the colonoids used in this study are derived from different colonic segments. Interestingly, although proximal and distal colons have different functions in digestion, their differences in gene and protein expression are not as prominent. A previous single-cell atlas study (40) reported that, in the three colonic regions sampled, there are comparable cell type proportions of all colonic cell lineages. Another intestinal single-cell study (41) showed a moderate decrease in absorptive enterocytes and an increase in secretory enterocytes from proximal to distal colon. Similarly, a colon membrane protein profiling study (42) showed that the levels of major proteins did not vary substantially along the length of the colon. In contrast, the magnitude of differences in cellular profiles and response phenotypes we observed among the three donors far exceeds the variation explainable by colon segments. However, we cannot definitively exclude potential contributions from segment-specific factors. In addition, the three donors varied in their age, sex, and ethnicity, which further limits the generalizability of the identified response phenotypes. To verify these results, a larger study that includes donor lines derived from the same colon segment with similar demographic characteristics and genetic backgrounds should be conducted. Second, the HCO culture system is oversimplified due to the lack of innate and adaptive immune cells, which play crucial roles in pathogen clearance. Co-culture experiments that incorporate immune components will help capture a more comprehensive profile of host-pathogen response. Nevertheless, considering the striking differences we observed across donors in this simplified system, we are certain that when combined with additional immune cells, this model will show additional complexity in donor-specific responses. Third, the 90-min infection time only provides a snapshot of the early acute phase of EAEC infection. A time-course study will be crucial to understand how donor-specific responses evolve over time and whether they predict disease outcomes after the bacteria establish stable colonization. However, the baseline differences that we observed are still striking regardless of the length of pathogen exposure. Fourth, our study is limited to the host transcriptional response to one single EAEC strain. Due to the diversity of EAEC virulence factors and their associations with different clinical diarrheal symptoms (12), it is difficult to generalize our findings to other EAEC strains, particularly those isolated from patients. Future studies that examine multiple EAEC isolates and other enteric pathogens are important in determining whether the host response patterns we observed (hyperresponsive, intermediate, and non-responsive) represent strain-specific adaptations or more general host defense mechanisms. Lastly, since our study is based on transcriptional level responses, it lacks verification for protein-level heterogeneity in cell composition, gene regulation, and cell-cell communication. Future investigations should integrate complementary approaches, including single-cell proteomics and immunofluorescence imaging of cell subtype markers and key signaling molecules. These studies will be crucial for confirming whether donor-specific transcriptional signatures correspond to functional protein differences that impact infection outcomes. Despite these limitations, our work serves as a proof-of-concept that human organoids combined with single-cell analyses can reveal profound host heterogeneity in genetic regulation and microbial infection response. It provides a framework for more systemic future studies that aim to discover donor- and cell-specific genes and pathways that can minimize infection-induced damage.

## MATERIALS AND METHODS

### Human colon organoid culture

HCOs from healthy adult donors were cultured as previously described (19). Briefly, HCO monolayers were generated from 3D enteroid cultures derived from healthy colon biopsy specimens (Baylor College of Medicine IRB: H-49668). 3D colonoids were maintained in Matrigel and subsequently dissociated into single cells for seeding onto Matrigel-coated chambered slides (Greiner Bio-One, 543979). Cells were cultured in complete medium with growth factors and ROCK inhibitor Y-27632 (Sigma, Y-0503) for initial attachment. After 24 h, the medium was replaced with differentiation medium, and cells were differentiated for 4 days before infection.

### Bacterial culture and infection

EAEC prototype strain 042 (serotype O44:H18) was cultured overnight in LB broth at 37°C with aeration, then sub-cultured 1:20 in HCO differentiation media for 2 h to obtain log-phase bacteria prior to infection. Day 4 differentiated HCO monolayers were then infected with EAEC 042 diluted in differentiation media at MOI = 10 for 90 min at 37°C in a humidified incubator. Mock-infected HCO monolayers were treated with differentiation media without bacteria instead.

### Bacterial enumeration

Infected HCO monolayers were washed with PBS and then dissociated using 100 µL/well of 0.05% trypsin-EDTA (Gibco, 25300-054). Cells were thoroughly dispersed by pipetting, diluted in PBS, and drip-plated onto LB agar. Plates were incubated overnight at 37°C, and colonies were counted the following morning.

### HCO monolayer collection for single-cell RNA sequencing

HCO monolayers on chamber slides were washed with PBS containing 5 mM EDTA and dissociated with 200 µL/well Accutase (STEMCELL Technologies, 07920). Cells were mixed using wide-bore pipette tips and incubated at 37°C for ~55 min until fully digested. Wells from the same HCO donor line and treatment were pooled, neutralized with fetal bovine serum, and diluted with 1 mL cold differentiation medium with ROCK inhibitor Y-27632. Then the cell suspensions were centrifuged at 300 × *g* for 5 min at 4°C, resuspended in 100 µL differentiation medium with Y-27632, and passed through Flowmi Cell Strainers (Millipore Sigma, BAH136800040). The resulting single-cell suspension was adjusted to 1,000 cells/µL for sequencing.

### Single-cell RNA sequencing

Single-cell gene expression libraries were generated at the BCM ATC Single Cell Genomics Core using the Chromium Single Cell 3′ v3.1 kit (10x Genomics). Briefly, HCO single cell suspensions were combined with reverse transcription reagents, Gel Beads containing barcoded oligonucleotides, and oil on a Chromium X instrument (10x Genomics) to form Gel Bead-In-Emulsions (GEMs), enabling barcoded cDNA synthesis from each individual cell. Subsequently, the GEMs were disrupted, and cDNA from every single cell was pooled. Following cleanup using Dynabeads MyOne Silane Beads, cDNA was amplified by PCR. The amplified cDNA was then fragmented, end-repaired, A-tailed, and ligated with sequencing adaptors. Final sequencing libraries were generated by amplification.

### scRNAseq processing and analysis

The 10x Genomics Cellranger software (v6.0) was used to demultiplex cell barcodes and map sequencing reads to the human reference genome (GRCh38). The resulting UMI counts matrix was further normalized using Scanpy (v1.8.1), where cells with UMI <500 were removed. Doublets were detected using the Scrublet (v0.2.3) package with an additional filtering removal of single cells containing greater than 8,000 genes detected. Since the samples were derived from different patients, batch correction was performed using BBKNN (v1.6.0) to account for technical variation. The dimensionality of the data set was reduced using principal component analysis, cells were clustered based on the Leiden method, and visualized by UMAP plots using Scanpy. Cell type annotation was accomplished using a combination of FindAllMarkers and FindTransferAnchors functions in Seurat (v5.0.3) along with published single-cell annotation (40, 43). Differentially expressed genes between infected and uninfected cells within each cluster and within each donor were identified using the FindMarkers function along with the Mann-Whitney Wilcoxon test, where the significant threshold was FDR <0.1 and absolute fold change >1.25. GSEA was performed using the Hallmark collection and a significance threshold of FDR <0.25. To identify developmental stages, pseudotime trajectory analysis was performed using the slingshot R package (v.2.10.0) without setting a starting cluster (PMID: 29914354). Inference of cell-cell communication was determined using the CellChat R package (v.1.6.1) and the CellChatDB of human ligand-receptor interactions. Cell lineages and pseudotime were inferred using the Slingshot R package (v.2.10.0).

## Data Availability

The single-cell sequencing data are publicly available at the GEO repository with accession number GSE335941.

## SUPPLEMENTAL MATERIAL

Supplemental material msphere.00758-25-s0001.pdf Supplemental table and figures.

ASM does not own the copyrights to Supplemental Material that may be linked to, or accessed through, an article. The authors have granted ASM a non-exclusive, world-wide license to publish the Supplemental Material files. Please contact the corresponding author directly for reuse.
