# Supplementary material for: Individual host variation in single-cell responses to enteroaggregative Escherichia coli infection modeled with human colon organoids
Source: mSphere. 2026 Aug 11;11(9):e00758-25. doi: 10.1128/msphere.00758-25 (PMC13621869; doi:10.1128/msphere.00758-25)
Supplement: Supplementary file 1 [file msphere.00758-25-s0001.pdf]

SUPPLEMENTAL MATERIAL

Supplementary Table 1. Donor and Sample Information

Donor	Age	Sex	Race/Ethnicity	Colon Segment	Infection	Cell Count (Total)	Cell Count (Post Filtering)
X	74	Female	Caucasian	Ascending Colon	Mock	2128	1286
					EAEC 042	3463	2300
Y	24	Female	African American	Unknown	Mock	10474	6258
					EAEC 042	13503	10463
Z	40	Male	Caucasian	Transverse Colon	Mock	11758	8874
					EAEC 042	13250	10170

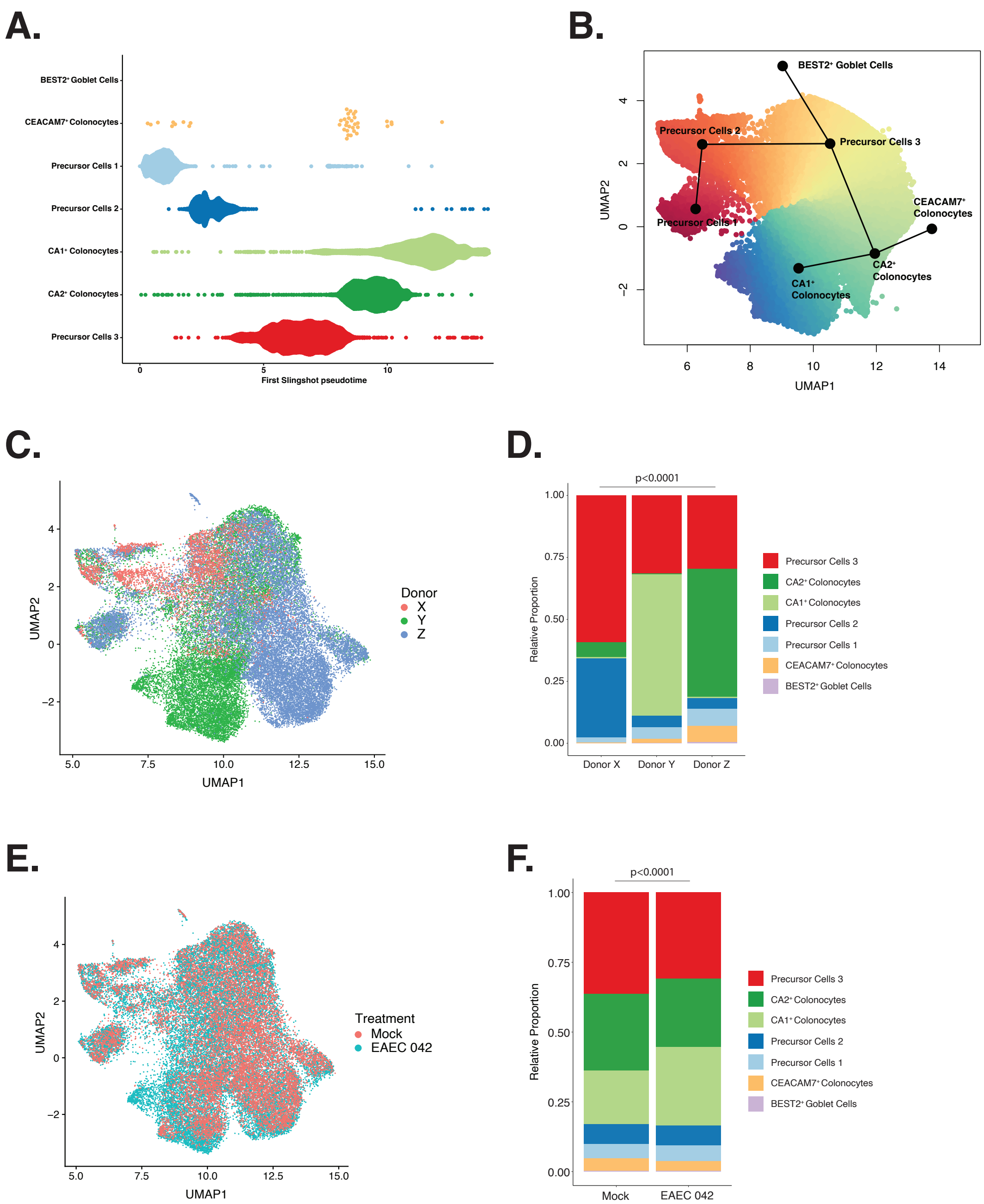

Supplementary Figure 1. Cell type clustering and trajectory analysis

(A) Violin plot showing Slingshot pseudotime for each cell cluster.

(B) UMAP showing Slingshot pseudotime trajectory analysis.

(C) UMAP showing all cells separated by donors.

(D) Bar plot depicting relative proportions of each cell cluster for each separate donor.

(E) UMAP showing all cells separated by infection condition.

(F) Bar plot depicting relative proportions of each cell cluster for mock- or EAEC-infected conditions.

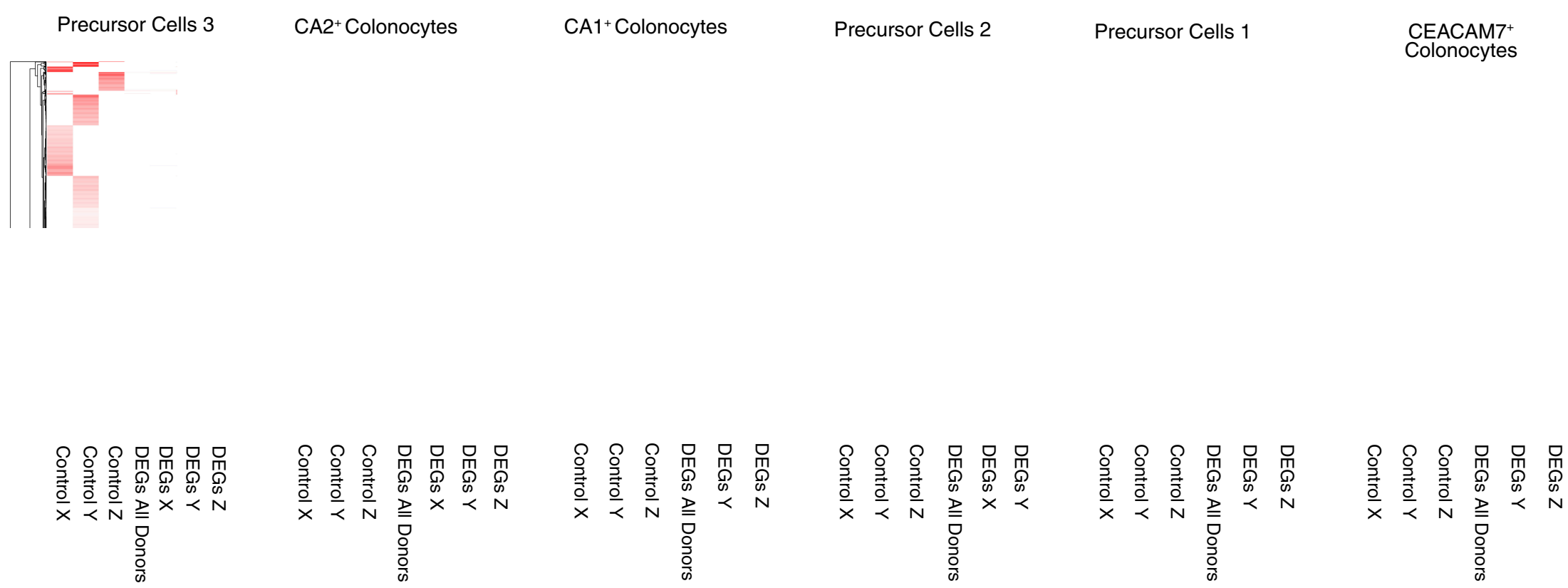

Supplementary Figure 2. Heatmaps showing markers of mock-infected controls and differentially expressed genes (DEGs) induced by EAEC 042 infection.
